# Supplementary material for: High-throughput screening of the effects of 90 xenobiotics on the simplified human gut microbiota model (SIHUMIx): a metaproteomic and metabolomic study
Source: Front Microbiol. 2024 Feb 20;15:1349367. doi: 10.3389/fmicb.2024.1349367 (PMC10912515; doi:10.3389/fmicb.2024.1349367)

Figure S1: Concentration of sulfasalazine over the course of seven days during the screening process. Biotic samples comprised SIHUMIx exposed to 1µM sulfasalazine for 24 hours, while abiotic samples consisted of CIM media and 1µM sulfasalazine.

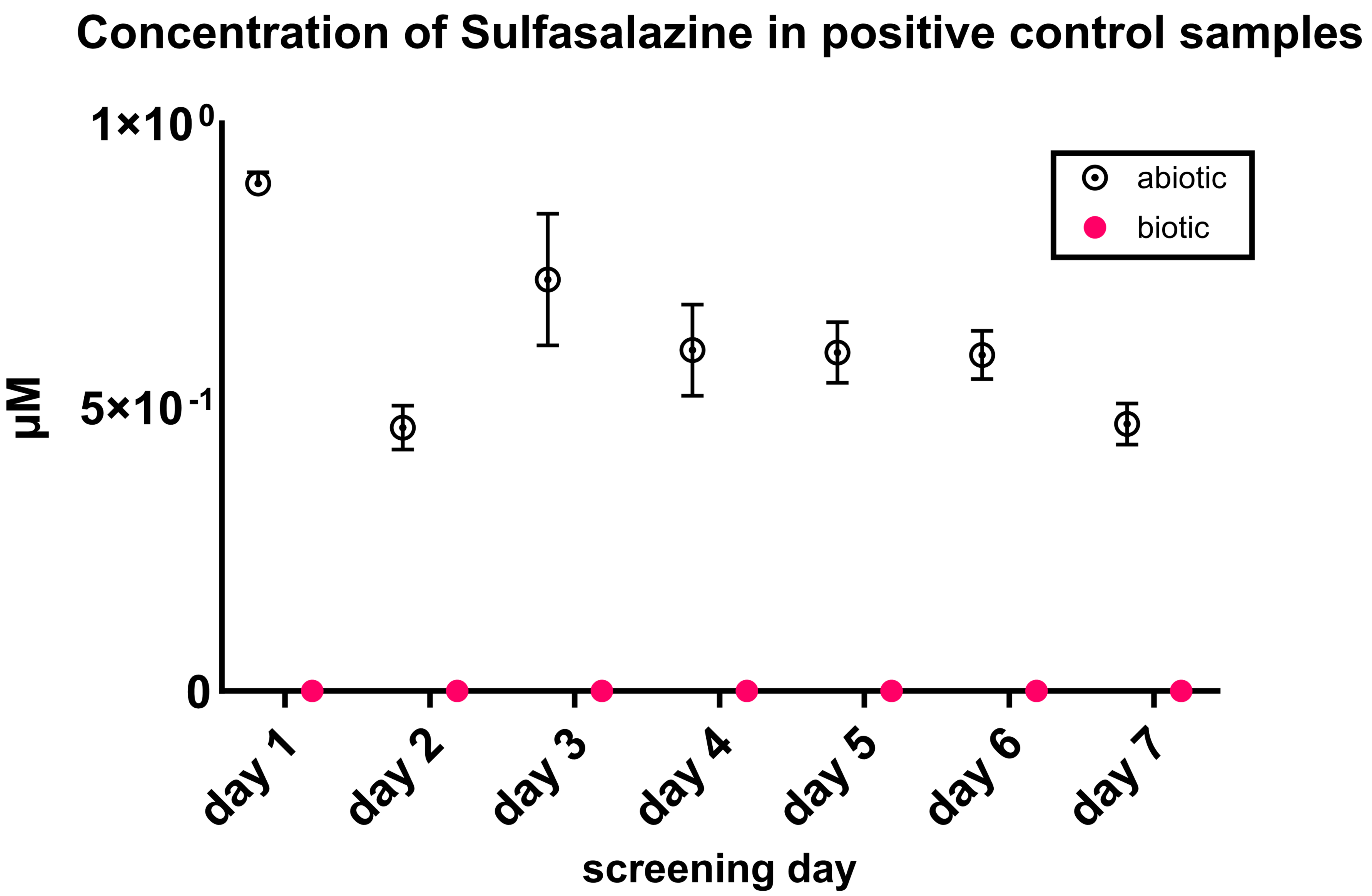

Supplement: Supplementary file 8 [file Image_1.pdf]
