# Supplementary material for: High-throughput screening of the effects of 90 xenobiotics on the simplified human gut microbiota model (SIHUMIx): a metaproteomic and metabolomic study
Source: Front Microbiol. 2024 Feb 20;15:1349367. doi: 10.3389/fmicb.2024.1349367 (PMC10912515; doi:10.3389/fmicb.2024.1349367)

Figure S5: Impact of food additives, and dyes on the metabolic pathways of SIHUMIx. The pathway intensity was measured by metaproteomics and is displayed as log_2FC for food additives and dyes which affected at least the intensity of one pathway compared to the control. Statistically significant effects (P_{adj} <0.05) are highlighted by asterisks

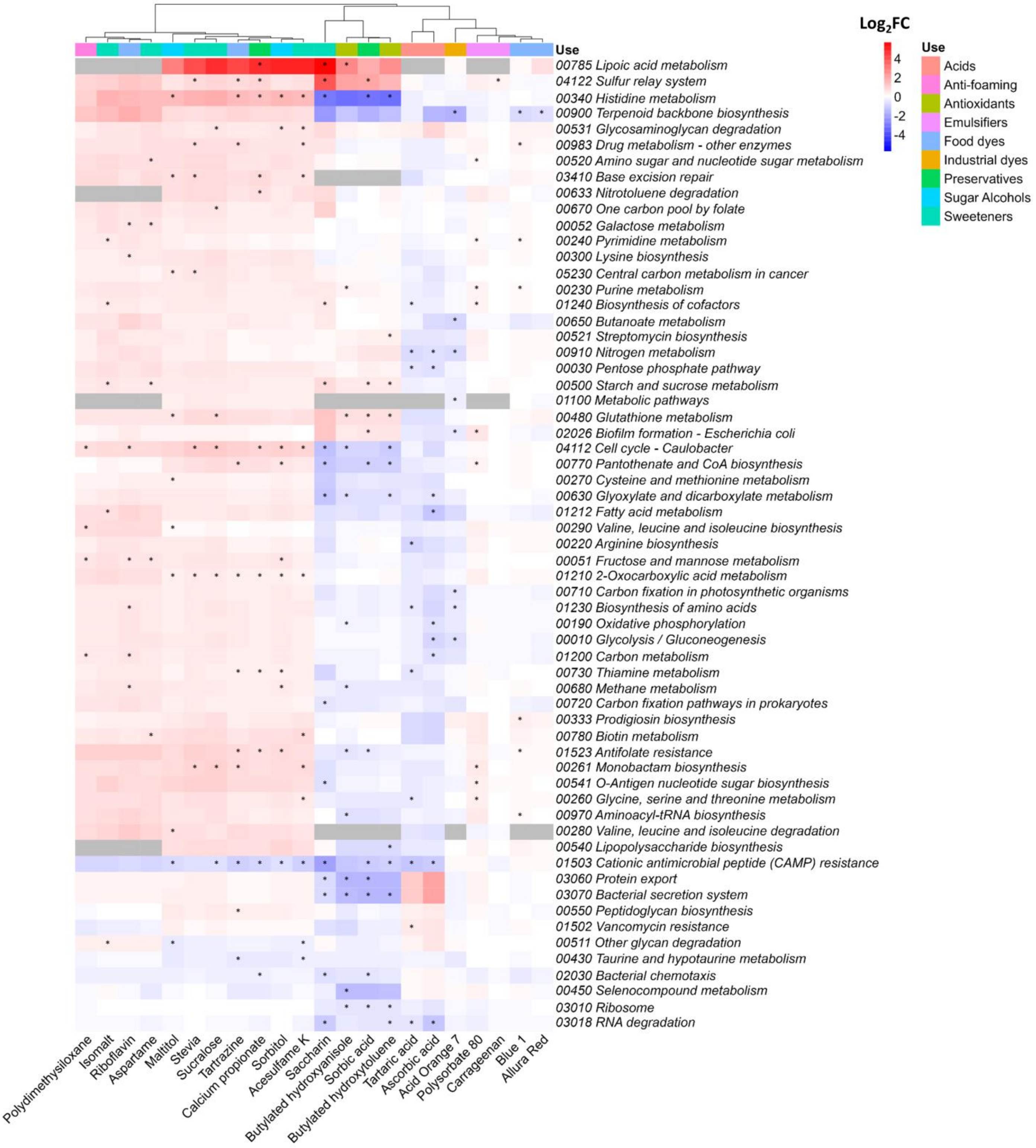

Supplement: Supplementary file 12 [file Image_5.pdf]
